# Abstracts &c.

**Published:** 1889-02

**Authors:** 


### ABSTRACTS AND GLEANINGS.

**Pneumonia.**—I was called to see a case, seven or eight miles from my office, a man, I presume his age was about twenty-two years. He had then been very ill for seven or eight days, expectorating almost pure blood from the second day of the attack. He had tried two other physicians, but growing no better he swapped them for me. When I arrived I found, by auscultation, that almost the whole of the right lung was consolidated or hepatized, as I could get no sound through the stethoscope, and on percussion there was a complete dullness. The left lung was clear. Cough very severe, so much so that he had rested very little since he was sick.

As I have before stated, he was expectorating almost pure blood, and his respiration was rapid and difficult; considerable pain throughout the right side and under the right shoulder blade. His temperature was 103½°, skin very dry and harsh, tongue had a dark brown pasty coat, red at tip and edges. Urine quite high colored, depositing a pinkish sediment upon the sides and bottom of the vessel. In fact, he was a very sick man and he believed, with his family, that he had lain down to die.

Well, the first thing I did was to rid my patient of the notion, and infused him with courage, telling him that he would get well—that he *must* get well, etc. I then placed over the seat of the disease a cantharides plaster, with orders that it must remain there until it raised a good blister. I then took an ounce of powdered asclepias tuberosa and had one pint of boiling water poured upon it, and ordered him to drink two ounces of this tea every hour till he perspired freely, then every two or three hours, as needed. I then prescribed the following for the fever, to be taken with a five-grain capsule of quinine:

| | | |
|---|---|---|
| ℞ | Tinct. Aconiti . . . . . . | 30 drops. |
| | Ext. Gelsemii fl. . . . . . . | 50 drops. |
| | Spts. Nit. Dulc. . . . . . . | 2 drachms. |
| | Syr. Simp. q. s. ad. . . . . | 10 drachms. |

M. Sig.: Shake well, and take a teaspoonful every three hours, with a five grain capsule of quinine, until four doses are taken, then every four hours.

For the cough I prescribed the following:

| | | |
|---|---|---|
| ℞ | Ext. Prun. Virg. fl. . . . . | 3 drachms. |
| | Ext. Glycyrrhizæ Pulv. . . . | 60 grains. |
| | Tinct. Lobeliæ . . . . . | 1 drachm. |
| | Morphiæ Sulph. . . . . | 2 grains. |
| | Syr. Simp. q. s. ad. . . . | 10 drachms. |

M. Sig.: Shake the bottle well before taking, and take a teaspoonful every two or three hours, as required.

For the great dyspnœa, I ordered an inhalation of one part vinegar and four parts water, heated to boiling, and used every two or three hours. This relaxed the lung and did much good.

I ordered egg nog given every three hours, alternated with chicken broth, and instructed them to give him butter-milk, diluted with water, when he wished to quench his thirst.

I had a jug of hot water placed to his feet, and ordered the room to be kept moderately warm, night and day, never allow it to get cold.

I returned to see him the next day and found his cough almost gone, expectoration with very little blood, temperature 100°, perspiration normal. Blister well drawn, respiration less difficult, tongue not much better, but not quite so red at point and edges.

The improvement was marked, and my patient was much more cheerful, as he suffered no pain, having also slept well during the greater part of the night. I ordered a dose of castor oil given as he had had no stool since I first saw him. I stopped the tea, as he was perspiring sufficiently. I also stopped the cough mixture, advising the nurse to give no more unless the cough should again become severe. The fever mixture and quinine I continued every five hours, and the egg nog, chicken broth, and boiled milk was to be given alternately every three hours.

I, being busy, did not see him again for two or three days; when I returned I found he was entirely rid of fever and every symptom improved, and I discharged him as convalescent. This case is given only as a sample of how I treat the majority of my pneumonia cases when they have a good constitution.

Pneumonia is an active disease and must be treated actively at its incipiency. I have used the sweating treatment for fifteen years and over, and I have but little need to complain.

Pneumonia is usually produced by sudden cold and a transmission of the blood from the capillary system into the lungs, and when we can get up a good perspiration we relieve, and by using plenty of quinine and stimulants we can bring them through all right.

DR. J. P. BAIRD, in *Medical Brief.*

Dr. I. M. Snow, (*Buffalo Med. Jour*,) writes: As chorea generally terminates in complete recovery, it is often asserted that direct medication is of no use. As a matter of fact, if a choreic child be taken from school and put to bed, the choreiform movements will diminish in violence, and the disease will commonly run a moderate course, but many cases do not improve under this expectant treatment, and may be greatly helped by well-directed therapeusis. It is the almost universal testimony that arsenic is directly beneficial, especially with children who have not the favorable environments of rest and quiet. With cases in which the movements are so violent as to prevent sleep and rest, Fowler's solution, diluted with glycerine, given hypodermically, is of extreme value. Cases of obstinate chorea of months' duration often improve under the hypodermic use of arsenic. The objection to this plan is that the pain of injection may lead to violent emotional attacks. In the absence of articular symptoms, anti-rheumatic remedies are of no use. Chloral often does great good in controlling the restlessness incident to the earlier stages, and inducing sleep. Strychnine is indicated if there be much paresis or muscular debility, and in chronic chorea iron is always valuable in the profound anemia which accompanies the disease· Sometimes spraying the spine with ether will quiet the movements. Dr. Barlow, of the Children's Hospital, was wont to emphasize the importance of keeping the bowels open and promoting the action of the skin by diaphoretics and by warm baths. He also advocated mild hydropathic treatment, douching the patient with lukewarm or cold water, the child being afterwards rubbed well and put to bed.

The last addition to the therapeutics of chorea comes from France. M. Legroux gives antipyrine in large doses—forty-five grains daily—to his cases, and reports astonishing success, curing his cases in from six to tweny-seven days. Chorea occasionally becomes so violent that the patient may throw himself against the bed, floor, etc., and the resulting injuries are exceedingly dangerous, numbers of patients dying from abscesses or septicemia. In these cases, it is well to secure the patient with bandages, or, better still, to lay him on a mattress on the floor.

Gymnastics have been highly extolled by M. Sée, but English and American authorities have not had the same success which M. Sée achieved. City children are greatly benefitted by frequent drives, and obstinate case will often very quicky convalesce when the little patient is shifted to some seaside resort.

**Medical Treatment of the Morphine Habit.**—When the tongue is loaded, the conjunctiva a little yellow, and the appetite indifferent, usually prescribe the following mixture, with good effect:

| | | | |
|---|---|---|---|
| ℞ | Acid. nitrici dilut., | . . . . . . | ʒ ij |
| | Tinct. nucis vom., | . . . . . . | ʒ iss |
| | Liq. taraxaci, | . . . . . . | ʒ iss |
| | Infus. aurantii comp., | . . ad . . | ℥ vj M. |

Dose, one-twelfth part two or three times a day, in wineglassful of water.

This mixture seems to be specially suitable for the sufferers from the morphine habit. It reduces the craving, and it favors the digestion of food.

The question is usually asked: Is there an antidote for the morphine, any drug which destroys the craving for it? The answer, I am sorry to say, is negative so far. I have thought that in a recent case I have seen some good from the administration of belladonna, but I must wait for more light before I venture on any statement of a definite kind.

One word more as to the endeavor to stop the habit by immediate control and systematic withdrawal of the narcotic from the habitué. Presuming that the habit has been quite recently acquired, I do not dispute that the attempt may be made with a chance of success. If made, however, it must be systematically and thoroughly done, under the most rigid medical supervision. There must be no playing with the trial. The strictest watch must be sustained, and the symptoms, always urgent, which follow upon the withdrawal must be met as they arise, with firmness, kindness and judgment. Moreover, when the symptoms have passed away, medical supervision, unbroken, must be kept up for three months at the shortest. All this demands residence in a house where every convenince for the treatment is at hand, and where a trusted and resolute physician is always at his post.—*College and Clinical Record.*

**Precautions as to Hypodermic Medication.**—The following precautions are worth remembering: Solutions of the alkaloids soon decompose and should therefore be freshly prepared. The smallest size of each tabloid given is the one generally preferred. The dose, hypodermically, is always less (the proportion varies) than by the stomach. Great care should be taken not to throw a medicament into a vein. Total collapse might ensue from injecting air into a vein. Absorption of an injection over the temple or chest is twice as rapid as elsewhere. The prick of the hypodermic needle in the chest has been followed by instant death. Syncope may follow an injection, if the patient does not remain quiet and lying down. For safety and freedom from pain, an injection should be made in the outside of the arms or thighs, or in the abdomen or back. Injections should not be made over bony prominences, or into inflamed or tense tissues. Mercury, ergot, etc., are best injected into the muscles, as in the nates. It is not usually considered safe to repeat an injection (as of morphine) for twenty or thirty minutes.—*Hospital Gazette.*

**The Combination of Sodium Chloride and Corrosive Sublimate as an Antiseptic.**—Lubbert and Shneider, after exhaustive experimentation, decide that sodium chloride, added to corrosive sublimate solutions, accomplishes the same results as do the addition of acids, and yet is free from many of the disadvantages of the latter. Common salt, when added to sublimate solutions increases the solubility of the mercury and prevents the precipitation of an albuminate when albuminous substances are present. A doule salt results, which is free from any irritant action upon tissues. They also recommend the addition of glycerine to sublimate dressings, as it renders them more smooth and increases the adhesion of the sublimate and gauze. Their conclusions are: that sublimate and gauze dressings are asceptic; that the reduction action of organic substances is reduced to a minimum, and hence the dressings longer retain their proportion of sublimate; further, that the addition of glycerine makes a smoother dressing; that the presence of the gauze is much increased by the presence of sodium chloride and glycerine; and that, owing to their ready oslubility, the combined salts are capable of destroying bacteria in the presence of albumens.

The authors found that corrosive sublimate gauze dries much more quickly if a little alcohol is added to the solution, but care must be taken that not too much is added, as in excess alcohol precipitates the chloride of sodium from its combination with the mercurial salt.

Therefore, the following solution is recommended for dressings:

| | | |
|---|---|---|
| ℞ Hydragrg. bichlor., | . . . . | 3 parts. |
| Sodii chlo., | . . . . . . | 100 " |
| Aq. destil., | . . . . . . | 600 " |
| Glycerin, | . . . . . . | 100 " |
| Alcohol, | . . . . . . | 200 " |

Dissolve the chloride of sodium in water, filter, then add the sublimate, and when it has completely dissolved add the alcohol. Dressings will usually absorb one and a half times their wcight of this solution—*Centralblatt fur Bacteriol*, No. 11, 1888.

**The use of Pilocarpine in Middle-Ear Diseases.**—*Ed. N. Y. Med. Record*, September 29:—The treatment of syphilitic and other affections of the internal ear by means of pilocarpine was recommended several years ago by Politzer. He stated that the drug was of benefit only in recent cases of these diseases, and that if no results were manifest after the lapse of eight or ten days its employment should be discontinued. He also asserted that dry catarrh of the middle ear, among other affections, was not benefited by pilocarpine. The therapeutic effect of the drug has been ascribed to the resorption or inflammatory exudation in the internal ear as a result of the increased action of the sweat and salivary glands.

In the *Archives of Otology* for June, 1888, Dr. W. Kosegarten, of Kiel, reports a few cases of aural disease treated by subcutaneous injections of pilocarpine, which would seem to show that the field of usefulness of this remedy is less restricted than Politzer, and others after him, have supposed. This author was more persistent in his use of the drug, not abandoning it after eight or ten applications, and he was rewarded by obtaining good results, not only in chronic labyrinthine disease, but in dry catarrh of the tympanum. He made it a condition that the patients should submit to a course of treatment of at least six week's duration, injections of one-sixth grain of pilocarpine being made daily, when possible. If the treatments were well borne, and if any improvment were perceptible at the end of this time, the injections were continued, and unusually with benefit.

His observations on the cases so treated led him to the belief that the favorable effect was due to a more active circulation in the tympanum, with consequent absorption of the inflammatory products and increased elasticity of the sclerosed tissues and adhesions. "It seems evident," he says in concluding his report of these cases, "that injections of pilocarpine also have a decided influence upon the mucous membrane of the middle ear, and therefore, in chronic processes of the middle ear accompanied by affections of the labyrinth, this remedy acts not only upon the internal ear, but that it can also exert a favorable influence upon the middle-ear process. By means of returning hyperæmia, which may even cause exudation, there ensues pliability of the sclerosed tissues and moistening and softening of adhesions, and in this way the unyielding conducting apparatus again becomes more capable of vibrating; where exudations had become deposited their absorption was brought about, as could be observed in the membrane tympani of a patient. The reason Politzer found the remedy inefficient in these affections was because he discontinued treatment too soon, for in these chronic processes we cannot accomplish anything except by long-continued action."—*Epitome.*

### A POSSIBLE SUBSTITUTE FOR TRACHEOTOMY AND INTUBATION IN CERTAIN CASES.

By Dr. Edgar Holden, of Newark, N, J.

*N. Y. Med. Record*, September 22—Under this modest heading Dr. Holden read before the American Laryological Association an excellent paper descriptive of a new and safe method of overcoming laryngeal obstruction. A short description was first given of its numerous causes, and of the sudden emergencies that call for the utmost haste in giving free access to the lungs as the only means of saving life. A long list of recorded mistakes, accidents, and dangers attending intubation was accompanied by a somewhat similar record in the history of tracheotomy.

The ingenious instrument he presented is obviously so designed as to render most of thcse unfortunate complications impossible, while operating with greater ease and rapidity. Thin silver blades are introduced through the crico-thyroid membrane and passed between the vocal cords. These are separated by pressing together the two short external extremities, which are at right angles to the rest of the blade and united to each other by a hinge where they rest in the wound. Pressing the other ends together separates the inner blades between the vocal cords.

Advantages: Easy of introduction and removal; cannot be displaced; breathing through natural passages; no interference with swallowing, etc. Also well adapted for removal of foreign bodies in the trachea.

A drawing was next shown of a very long silver tracheotomy tube, designed by A. Gouguenheim, of France, the lower end having flexible spiral. Introduction easier and injurious pressure avoided—*Epitome.*

**The Nasal Origin of Whooping-Cough and its Treatment.** —An important chapter in pathology has been created within the past two years, namely, that which refers to the reflexes of nasal origin. Since 1873, when Voltolini showed the relations subsisting between asthma and mucous polypi of the nose, several memoirs upon nasal neuroses have been published in Germany, America, and France. Among them is that of Hack, of Freiburg which was published in 1883, and demonstrated that certain nasal lesions, especially hypertrophy of the lower segment of the mucous membrane, could cause by reflex action: (1) asthma and hay fever; (2) laryngeal spasm: (3) cough; (4) certain forms of neuralgia, especially hemicrania; (5) redness and swelling of the skin of the nose; (6) attacks of vertigo: (7) attacks of epilepsy; (8) anomalies of secretion. These different neuroses disappeared after destruction of the cavernous bodies of the nose by means of the galvano-cautery. Michael, of Hamburg, published in 1885 his views concerning the reflex-nasal origin of whooping-cough, and the method of treatment which he had adopted. He used neither chromic acid, nitrate of silver, nor the galvano-cautery; he objected to naso-pharyngeal douches as difficult of execution in children, and liable to produce otitis media. He found that the insufflation of suitable powders accomplished the desired end, and experimented with tannin, boric acid, iodoform, and benzoin. The last of these was the most serviceable of any.

Michael treated fifty children by the method of nasal insufflation, the result being a diminution in the number and violence of the paroxysms after the first treatment. In a number of cases the disease was limited to its first stage by this plan. Only one insufflation daily was practised, the powder being injected during expiration to prevent its entering the mouth or larynx. Michael's theory is that whooping-cough is a parasitic disease, and that the insufflation powder acts upon the infectious element and thus produces a curative effect. Guerder has also employed this method, using a powder composed of equal parts of boric acid and roasted coffee. The results with him also were that the paroxysms diminished in frequency and in intensity, and also that the general condition began at once to improve. Bochem treated sixteen children successfully with insufflations of a powder composed of three parts of muriate of quinine to one of gum-arabic. Lubinski, Stoerk, and Ziem have been equally successful with, and are equally favorable to this method of treatment. —*Archiv. Pediac.*

**Opium** acts very much like alcohol in the human system, both being primarily stimulating and subsequently narcotic; and both are at once narcotic in a large dose. Opium causes confused ideas, reverie, pleasurable delirium, and voluptuous listlessness; a sort of a "fool's paradise," from which, however, the patient can generally be arroused to transact business, unless "too far gone." Unfortunately, he experiences no after-inconvenience from an occasional indulgence in smoking opium; but its habitual use has most disastrous effects.

**Treatment of Anal Fissure Without Operation.**—According to a Paris letter (*Gaillard's Med. Jour.*, September, 1888), Dr. Gregney believes that he has discovered a simple, painless, but effectual method of curing all fissures of the anus without resorting to operation. It consists in first securing a thorough evacuation of the bowels every morning, and then introducing between the lips of the fissure a few shreds of lint saturated with a solution of chloral, 1 to 50. This lint is left in *situ* until the evacuation of the rectum next morning carries it away, when it is replaced by shreds similarly treated. These dressings are repeated daily until the fissure disappears, which is usually about the tenth day of treatment.—*Satellite.*

**Eruptions due to Antipyrine.**—Spitz has collected references to fifty-two cases of antipyrine eruptions. In forty-one cases the eruption is described as rubeola-like, in four urticaria form, in others erythema-form, or papular. The antipirine was given in thirty cases of typhoid fever, in eight cases of phthisis, in two cases of articular rheumatism. In the other cases the concurrent disease was not reported. The seat of the eruption varied greatly in different cases, but was usually on the extensor surface of the limbs. The average duration of the eruption was four to eight days.—*Ann. de derm. et de Syph.*, vol. ix, 1888, p. 192; from *Theraputische Monatshefte.*

**Thuya Occidentlalis in Epithelioma of the Larynx and Pharynx.**—Baratoux, of Paris, reports encouraging results in cancerous growths, especially epitheliomata, from the use of thuya, administere internally and locally. He tried the tincture and alcoholic extract of both *thuya occadentlis* and *thuya orientalis* but the latter in no way resembled the former in therapeutic value.

He administered daily doses of twenty drops of the tincture, which were gradually increased until forty-five to sixty minims were reached. At the same time the diseased parts were painted with the pure tincture every second or third day, sometimes every day. If the trouble be located in deep parts, such as the post-nasal space, he recommends a spray composed of the following ingredients:

| | |
|---|---|
| ℞ Tinct. of thuya occidentalis, . . . | gr. lxxv. |
| Distilled water, . . . . . . | ℥iij. |
| Glycerine, . . . . . . . | ʒiiss. |

The effects of this medication are very rapid, eight days at most sufficing to cause diminution, and even disappearance, of the fetidity of the mucoid and purulent expectoration. The secretions also become less abundant and about the twentieth day one may observe marked changes in the volume of the vegetating mass.

A remarkable fact is that in papillomata of the nose and ear, as was the case with Baratoux, a complete cure may be expected, while, if the structure of the tumor be mixed, the neoplasm diminishes in volume, but cannot be made to disappear completely. Sacromata and carcinomata do not seem to be influenced at all by the drug.—*Journal de Medecine de Paris*, May 13, 1888.

**Strychnia an Antidote for Morphine Poisoning.**—Mr. C. took at four o'clock p. m., while drinking, six grains of morphine, and in a short time was sound asleep and could not be aroused. In half an hour afterwards an effort was made to clear the stomaeh by vomiting, without effect, and other means to arouse the young man proved as fruitless as the emetics; the man slept in spite of everything. I saw him at eight o'clock p. m., four hours after he took the morphine, and found him with all the symptoms of morphine poisoning, pulse slow, and breathing very slow and stertorous. In fact, he was in a profound coma, and it was concluded by all that he must die. His extremities were cold and blue, no muscular resistance could be excited by any means. I gave him one-thirtieth of a grain of strychnia hypodermically, and in a short time his pulse and breathing improved greatly, and in ten or fifteen minutes he opened his eyes when violently shaken and sharply spoken to. At this time efforts were made to wake him, after which time, say about one hour, I again injected him with one-thirteenth of a grain of strychnia, and in a short time was surprised to see the magical effect of the remedy. In a few minutes more the man was conscious. During the interval between the injections of strichnina he took five grains of carbonate of ammonia every half-hour. He madc a good recovery.

I consider this a grand victory, as I had two poisons to encounter, morphia and alcohol. I do not know that this is a new idea, but it was new with me as I had never read of a cure of morphia poisoning treated with strychnia, and offer this to the profession. If it is not new, it may reach some one who may have never heard of it before. —*Dr. Fletcher in Leonard's Med. Jour.*

**Salix Niger.**—A sexual sedative of the highest order, administered in from half to one drachm doses, three times a day, exceeds in therapeutic power the action of the green root tincture of gelsemium and the bromides combined, without any deleterious effect whatever.

The fluid extract of the black willow, highly ozonized, is a tonic, stimulant, sedative, astringent and germicide. Its area of action is the genito-urinary organs of both sexes.

Our country, our climate, our civilization, our literature, predispose our youth to hyperemia of the lumbar portion of the cord which presides over the genitalia, and to such affections as nyphomania, satyriasis, onanism, seminal incontinence, ovaritis cystitis, prostatitis. Its action is most remarkable in relieving and allaying all irritation.

In natural emissions of our drained out young men, the drug is used with the most marked benefit. The pollutions cease entireiy, virile power and passion are not in the least diminished.—*Pacific Med. Rec.*

**WARTS.**—It is stated that warts may be cured by the internal administration of one to two drops of Fowler's Solution of arsenic three times a day for ten days.

**A Pleasant Purgative for Children.**—It is conceded, I believe, that castor oil would be the most popular purgative for children known, if it were not for its objectionable taste; that aromatic syrup of rhubarb is a most excellent purgative, and would be used almost to the exclusion of other preparations if the irritant after effect that follows its administration did not militate against its employment; and that cascara cordial from its desirable tonic properties would be the *sine qua non* only its proper rôle is as a tonic curative laxative, and is too uncertain as a purgative.

A friend kindly informed me recently that he employs the prescription given below with success. The combination pleased me. I have tried it, and it fulfills all indications (including palatableness) more satisfactorily than any purgative I have prescribed. It will serve equally well for grown up children:

℞ Castor oil,
Aromatic syr. rhubarb,
Cascara cordial, ää f ℥ j.

M. Sig.—Dose, one teaspoonful, or as may be needed.

DR. R. R. MITCHELL, in *Med Age.*

**Some Advantages of Membership in Medical Societies**—There is, perhaps, no other one thing that conduces so much to the advancement of medical science as does the active working medical society. Now and then we hear it said, "The profession is going wild over medical societies; they are getting to be entirely too numerous." Those who hold this view lose sight of the fact that compulsory attendance upon society meetings is a thing unknown. Every one will admit that it is possible for a physician to devote so much time and attention to society work as to seriously affect his private practice, but a smaller number of patients are lost in this way than by the stay-at home plan.

The *Maryland Medical Journal* says: "By the thoughtful, live physician, the question, 'does it pay to belong to a medical society?' will always be answered in the affirmative." This, however, is not apparent to many, as the renumeration, in dollars and cents, comes in an indirect way. The *Journal* continues thus:

"The amount of practical information which a physician may gain from the discussions of an active society is beyond all calculation."

Nor does the medical society teach him less about himself.

It gives him opportunity to compare himself with his fellows, to silently note the points in which he is deficient. It trains him to greater accuracy in the study of his cases, and greater care in their treatment, especially if from time to time he brings the more interesting ones among them to the notice of the society.

Here and there may be found an active, pushing city practitioner who is a member of no society, but this is a rare exception. Our best workers are society men.—*Weekly Medical Review.*

**Felons.**—Dr. Edison in (*Med. World*) sensibly remarks: I have no new remedy to propose, but I wish to emphasize the value of a very, *very* old one—*prompt*, *deep*, *effectual incision*. By this means, in a moment, a painful abscess is converted into little more than a simple incised wound, and thus untold relief is afforded.

The resort to topical applications and delay is presumed to be in the interest of conservatism and humanity, but the effort to avoid the direct issue is a mistaken forbearance, which often entails most serious consequences.

I am not of a sanguinary disposition, but there are a few simple operations that afford me such a feeling of satisfaction as I derive from this, when I see the pus "welling up" beside the knife. I know that I am doing good, and the patient never fails to appreciate it also.

If you are in doubt whether or not to operate, give the patient the benefit of the doubt by incising at once. You will never regret it.

**Albuminuria in Pregnancy.**—*First.* In all cases of pregnancy, not only should examination of the uterus be systematically made, but the eyes should be examined with the ophthalmoscope; since, in a large proportion of cases where eye troubles exist, the patients make no complaint of disorders of vision. Frequently such troubles can be detected with ophthalmoscope long before any disease of the kidney is shown in the urine.

*Second.* In uræmic amaurosis, without changes in the eye visible to the ophthalmoscope, even should the usual accompanying symptoms, a dizziness, nausea, and threatened convulsions, be absent, their supervention is soon to be anticipated, and the immediate induction of preamature labor is indicated, without waiting until the life, as well as the sight, of the patient is in danger.

*Third.* In neuro-retinitis the induction of preamature labor is not only justifiable, but urgently demanded. In some instances it is called for even in the earlier months of pregnancy.

*Fourth.* It is required in cases of eye trouble recurring in successive pregnancies, like Loring's.

A woman having once suffered in this way during pregnancy, the relationship of cause should be fully explained, both to herself and her husband.

Dr. Fryer (Missouri Med. Ass., *Kansas City Medical Index*, July) gives the details of a case of impaired vision in a pregnant woman, appearing in about the fifth month of gestation. Examined in the sixth month by the ophthalmoscope, there was found typical albuminuric retinitis without hemorrhages; the urine was loaded with albumen, diminished in quantity, of a specific gravity of 1028. There had been no symptom of kidney-lesion, no œdema of any part. Premature labor was advised, but deferred, with rapid decrease of vision to a meré perception of light, and continuous cephalalgia. Premature labor, however, was finally carried out. There were no convulsions during labor, and health finally returned, except that complete blindness followed. The retina became apparently normal in appearance, but optic atrophy was plain. Dr. Fryer quotes the conclusions of Dr. Howe from an examination of cases published after 1870, whence it appears that where the albuminuric retinitis appears prior to or during the seventh month of gestation, the rule is that the patient becomes blind, death often occurring from convulsions during labor.

According to the abstract of the *Centralblatt* of April, Dr. J. L. Thompson induced preamature labor in a woman in the eighth month of her pregnancy, who had became completely blind in nine days. The result was happy, vision—$\frac{20}{50}$ and $\frac{20}{30}$—returning in a few weeks. A second patient died during the first visit, and a third, who had become suddenly blind, died after preamature labor. Convulsions appeared in a fourth ten days before delivery, with permanent blindness.

The case of Dr. Wadsworth (*Boston Med. and Sur. Jour.* January 5) is of interest, as showing retinal detachment, with extensive choroidal changes. Visual deterioration began about the seventh month, preceded and accompanied by moderate œdema of the lids and legs. The urine was loaded with albumen, and contained hyaline and granular casts. Subcutaneous injection of pilocarpine did not seem to arrest the progress of the disease. Premature labor was induced about the eight month of pregnancy. The child lived. The hitherto extremely poor vision slowly improved, so that some nine months afterward it was $\frac{14}{30}$ and $\frac{14}{15}$. The retinal detachment was greatest in the right eye, in which it was quite general, but the replacement seemed at last to be perfect. The widespread pigment changes produced defects of vision, but the fields are reported as normal in extent. The white spots of the macula region disappeared.

A case of "puerperal uræmic amaurosis" is reported by Marcuse (abstract in *Lancet* of June 16th) that came on immediately preceding the delivery. There was a rapid convalescence and return of vision. —*Dr. Poorley*, *Acad. Med.*, *Med. News.*

**Treatment of Incontinence of Urine by Rhus Aromatica.** —*Ed. Boston Sur. and Med. Jour.*, November 8: Rhus aromatica has recently come into vogue in France as a remedy for incontinence of urine in atonic states of the bladder. According to *La Therapeutique Contemporaine*, Drs. Max, Burvenich, Numa, and Hamon have all tried it in this disorder, and have obtained signal successes where all other remedies have failed.

Max prepares a tincture by macerating 200 grammes of the bark in a 1,000 grammes of alcohol at 80°. The dose is from ten to fifteen drops three times a day. Max claims nine cures out of eleven cases. Burvenich obtained a cure in eleven out of thirty-three cases: ten of the remaining twenty-two were considerably benefited. The good effect of the medicine is not felt till the fifth or sixth day; sometimes satisfactory results are not perceived till at the end of three or four weeks.

According to Burvenich, Rhus aromatica constitutes a powerful tonic similar to nux vomica, for in a man aged seventy-nine years, affected with incomplete paralysis of the bladder without stricture of the urethra or hypertrophy of the prostate, rhus aromatica, in the dose of thirty drops a day, greatly facilitated micturition by giving tone to the bladder. Numa also asserts that it excites the unstriped muscles of the bladder as well as of the uterus and the rectum. He gives the tincture to children from two to six years of age in the doses of ten drops twice a day, and to older children in the dose of fifteen drops night and morning, The tonic effects do not last, for the paresis of the spincter vesicæ muscles returns when the medicine is discontinued.

Hamon has also testified to the efficacy of rhus toxicodendron in certain cases of incontinence of urine, with atony, in young persons. He gives twenty drops at bedtime in a little simple elixir, or other convenient menstruum. This use of rhus toxicodendron is not new.—*Epitome.*

Treat cracked nipples by covering the fissures with india rubber or caoutchouc dissolved in chloroform. This forms a pellicle, which protects the fissures against the infantile saliva.

Dr. Paul (Paris) has demonstrated that saccharine is a powerful antiseptic and destroyer of certain microbes, especially the bacterium germs and staphylococcus aureus. He prescribes it as a cleanser of the digestive tract in the following form:

| | | |
|---|---|---|
| ℞ | Saccharine, . . . . . . | gr. 80 |
| | Soda bicarbonat . . . . . | ʒ j |
| | Alcohol (40 per cent.) . . . . | ℥ iij |
| | Syr. menth. pip . . . . . | ℳ xx |

One teaspoonful in a wineglassful of water, thrice daily.

Saccharine in solution also forms an excellent mouthwash, quickly destroying the bacteria that infest that region;

**A Method of destroying Tatoo Marks.**—Dr. Variot has discovered a means of removing tattoo marks, a result hitherto reported to be difficult, and even impossible of achievement. He pours on the marked spot a concentrated solution of tannin, and works it into the skin by a series of pricks, just as in tattooing proper. A certain quantity of tannin is thus introduced beneath the skin. He then rubs the parts with nitrate of silver, and allows the solution of the salt to remain in situ until the prick marks show out as black points. The caustic is then wiped off, and the result is the formation of a black stain of tannate of silver. Inflammation is set up, and in the course of a fortnight scabs form, on the disappearance of which no trace is left of the original design, the only souvenir being a redish scar, which in time becomes less visible. Various other plans have been tried without success—scarification, the introduction of opaque powders and caustics into the skin, etc. In no case did he have to deal with troublesome suppuration, although if the area be large it is well to do a piece at a time.—*Med. Pres and Circular.*

The *Cincinnati Medical News* says that a physician whom the writer knew well died four years ago. He had cared for a large practice during forty years. At his death his house was found to be mortgaged at nearly its full value; he had no bank account, and his chattel mortgage was almost valueless—worth only a few hundred dollaıs—but of it all there was insufficient to pay his debts. An only daughter who previously had never known want, was compelled to trudge the country to sell books until weary and sick she laid down and died. Yet the doctor had lived most economically and had not a vice. No man possessed a purer character or was more upright. His only fault was that he kept his books so carelessly that he was easily defrauded by one inclined so to do. The moral is keep your books with the same scientific accuracy that you study your anatomy, and with the same diligence that you look after your patients. To love one's neighbor as one's self is all that is required by the highest benevolence. Get such account books as will reduce to a minimum the labor of keeping your accounts and then look after them every night seeing that all work performed is properly entered so that it will stand in a court of law should you die during the night and your books pass into other hands to-morrow for settlement. This is a little thing, but it hinges many great things. It is an irksome labor, but it is a duty which the doctor owes to himself, to his family and to his patients.—*American Lancet.*

**Dysmenorrhœa.**—The Paris correspondent of the *American Lancet* writes: Dysmenorrhœa in young girls is treated by Goubet (*Gaz. de Gynecolgie*), with iodoformized asafœtida as follows: Iodoform, gr. ½; ext. belladonna, gr. ⅛; asafœtida, gr. 1½. Make into one pill, which is to be taken six times daily a week before menstruation.

Galvanism in Acute Sub-acute Pelvic Inflammation, is the subject of a paper by Apostoli in *Nouveau Arch. de Obstetrics et Gynecologie.* Fever and inflammatory conditions do not necessarily preclude in his mind the methodical use of the constant current. Salpingitis and hydrosalpinx may be safely so treated if the tumor be close to the fornix. Suppuration is a contra indication to this mode of treatment.

Puerperal Mastitis is the subject discussed by Olshausen in the *Annals de Gynecologie.* He, with most of the new school has accepted the theory that mastitis is always consequent upon infection. Proper treatment includes strict antisepsis and the cleansing of the mouth of the infant. When the process of inflammation is superficial apply bladders of ice. If the fever continues to 36 hours suppuration is certain and incision becomes necessary. Drainage and dressing the wound antiseptically complete the treatment.

Antipyrine for after pains is the subject of a memoire by Riviere (*Gazette Hebdomadaire*). The author shows the advantage of antipyrine for diminishing the pain without modifying the contractions of the uterus and in consequence relieve the after-pains. It possesses a real superiority to opium which calms the pains and suppresses the contractions. The author has administered antipyrine *via* the stomach in doses of one gramme, and in this manner has in many cases decidly calmed the after-pains. He in some cases increases the dose to two grammes but that is the maximum and it is rarely necessary to pass it. When given in this dose the antipyrin is administered in divided doses, the one separated from the other by an interval of one hour.

Contagiousness of Vulvo-Vaginitus was the subject of a report by M. Olliviere made to the French Academy of Medicine (*Gazette Hebdomadaire*). He had observed the disease in five little girls who are being cared for the diseases of children. Five days after the arrival of two of these little girls affected with this disease the three others were successively contaminated. The transmission is made by the hand of the attendant, and the articles used in cleaning. After the adoption of antiseptic precautions it was not transmitted to a new case.

Double Placenta existing in a single pregnancy was the subject of an interesting specimen exhibited to his colleagues at the French Academy of Medicine by L. Guemot. This was a very rare specimen. There were two placental disks and each was supplied with its own vascular apparatus which emanated from the cord direct.

Utero-Ovarian Gout is considered by Dr. Mabboux in *Bulletin de Gen. de Therap.* and the following conclusions reached. 1. Gout may localize itself in the female genital organs during sexual life as well as after its cessation—there are metritis, vaginitis and vulvitis of gouty origin. 2. These outbreaks of genital gout may occur either during menstruation or in the dysmenorrhæic form, or in the interval. 3. Utero-Ovarian Gout is amenable to the same hydro-mineral medication as are other forms. 4. After the subsidence of all inflammatiory action the strong sodium bicarbonate waters are indicated in the non-anæmic and especially in the plethoric. 5. The cold calcic sulphate waters are beneficial in most cases because of their resolvent, sedative and tonic action. They may be made use of soon after the acute stage.

**Antipyrin as an Anodyne in Labor.**—In a preliminary note in the (Polish) *Wiadomosci Lekarskie*, No. 10, 1888, p. 289, Dr. F. Sielski, House Surgeon to the Lemberg (Lwow) Hospital (Galicia, Austria), states that he has given antipyrin in three cases of labor at full term, and in one case of abortion (fourth month). He came to the conclusion that "the drug in many regards is much superior to all other means which have been hitherto recommended for the relief of the pains of labor." He usually gave one gramme of antipyrin, and when necessary, repeated the dose every two hours. "The result was invariably excellent. A few minutes after a dose the pain ceased almost entirely, while the force of the uterine contraction did not decrease in the least." The patients felt only the pain accompanying the passage of the infant's head through the genital canal; "but even this pain was beyond all comparison less than in preceding labors.—*British Medical Journal.*

